# Sporoderm-broken spore powder of *Ganoderma lucidum* ameliorate obesity and inflammation process in high-fat diet-induced obese mice

**DOI:** 10.29219/fnr.v66.8745

**Published:** 2022-10-12

**Authors:** Bao Zhong, Feng-Lin Li, Jia-Yao Zhao, Yao Fu, Cheng Peng

**Affiliations:** 1 School of Food Engineering, Jilin Agriculture Science and Technology University, Hanlin Road, Changyi District, Jilin 132101, China; 2 School of Economics and Management, Jilin Agriculture Science and Technology University Hanlin Road, Changyi District, Jilin 132101, China; 3 Jilin People’s Hospital, Zhongxing Street 36, Jilin 132102, China; 4 School of Biological and Pharmaceutical Engineering, Jilin Agriculture Science and Technology University Hanlin Road, Changyi District, Jilin 132101, China

**Keywords:** sporoderm-broken spore powder of *Ganoderma lucidum*, anti-obesity, anti-inflammation, adipocyte, ameliorate, high-fat diet

## Abstract

**Objective:**

This study examined the anti-obesity and anti-inflammatory effects of sporoderm-broken spore powder of *Ganoderma lucidum* (SSPL) against obese mice fed with a high-fat diet.

**Methods:**

Four groups of C57BL/6J mice were randomly assigned to the following diets: control diet (CD); high-fat diet (HD); high-fat diet plus l-carnitine (HDL); and high-fat diet with sporoderm-broken spore powder of *Ganoderma lucidum* (HDG). They were subjected to 12 weeks of testing.

**Results:**

Supplementation with SSPL lowered weight gain caused by a high-fat diet and improved serum and liver lipid levels, and histological investigation indicated that the HDG group had a significant reduction in liver lipid deposits and adipocyte size in epididymal fat. SSPL administration decreased the expression of genes associated with inflammation and fat anabolism, including tumor necrosis factor-alpha (TNF-α), interleukin-6 (IL-6), interleukin-1β (IL-1β), peroxisome proliferator-activated receptorγ (PPARγ), sterol regulatory element-binding protein 1c (SREBP-1c), fatty acid synthase (FAS), acetyl-coenzyme A (CoA) carboxylase (ACC), and leptin. SSPL therapy raised the levels of PPARα, carnitine palmitoyl-transferase 1 (CPT-1), acyl-CoA oxidase1 (ACOX1), and adiponectin.

**Conclusion:**

In summary, SSPL protected mice against developing obesity caused by increased fat intake by regulating inflammatory factors and lipid metabolism. Our findings indicate that SSPL is a potentially beneficial healthy meal for treating obesity.

Obesity is a metabolic syndrome caused by an energy intake/consumption imbalance. Obese rising prevalence is alarming (1). Abnormal bodyweight overload is identified as a body mass index (BMI) more than or equal to 25 kg/m^2^, and obesity is characterized as a BMI higher than or equal to 30 kg/m^2^ based on WHO criteria (2). Clinical study has established a link between persistent low-grade inflammation of adipose tissue and being overweight or obese (3). Excess weight gain increases the chance of developing various diseases, the most serious of which are cardiovascular disorders and non-alcoholic fatty liver disease (NAFLD), all of which are linked with an increased risk of death (4). Anti-obesity medication, bariatric surgery, and lifestyle change are now used to treat obesity; however, there is inadequate evidence for the safety and efficacy of anti-obesity medicines and in post-bariatric surgery patients (5). Currently, research has focused on developing functional foods or nutraceuticals that can ameliorate obesity, such as edible fungus (6, 7).

*Ganoderma lucidum* is a medicinal fungus that has been used in China for centuries to promote health and longevity. Previous studies have shown that triterpenes and polysaccharides of *Ganoderma lucidum* (PGL) inhibit adipocyte differentiation and produce hypoglycemia effects, as well as are also used to prevent and treat various diseases such as cancer, hepatitis, and obesity (8). *Ganoderma lucidum* may decrease 3T3-L1 adipocyte development and lipid accumulation (9). Ganoderic acid A has been shown to ameliorate obesity, lipid accumulation, and insulin sensitivity in mice fed with a high-fat diet via modulating the sterol regulatory element-binding protein (SREBP) pathway (10). The major regulators of lipogenesis are SREBP-1c and peroxisome proliferator-activated receptor (PPAR). Downregulation of SREBP-1c and PPAR inhibited the expression of lipogenic genes such as fatty acid synthase (FAS) and acetyl-CoA carboxylase (ACC) (11, 12). Treatment with ganoderic acid A and PGL could improve NAFLD (13, 14). PGL has been demonstrated to have many pharmacological functions, such as anti-inflammation, antitumor, anti-obesity, and antidiabetic effects; for instance, PGL was able to prolong tumor-implant mice lifespan, either administered alone or in combination with cytotoxic and antitumor medicines (15, 16). Despite the fact that *Ganoderma lucidum* has been shown to have strong protective benefits in the prevention or treatment of a number of diseases such as cancer, NAFLD, and obesity, the mechanisms underlying the prevention of obesity and anti-inflammatory of *Ganoderma lucidum* remain undefined.

*Ganoderma lucidum* spore is a minuscule germ cell ejected from *Ganoderma lucidum* gills during its growth maturity period. It has been considered with high exploitable potential in healthcare products manufacture (17). *Ganoderma lucidum* spores contain all active ingredients of *Ganoderma lucidum*, and also the content of the major ingredients, including polysaccharides, and triterpenic acid is relatively higher and displays more prominent biological activities (18, 19). *Ganoderma lucidum* spore powder is the raw material for extracting *Ganoderma lucidum* spore oil, and also manufactured into microcapsules. In 2020, sporoderm-broken spore powder of *Ganoderma lucidum* (SSPL) has been included in the healthy food catalog in China, and the future research prospects are broad. However, there is, indeed, a paucity of literature on managing obesity and inflammation using SSPL. Consequently, we suggested that SSPL might have the prevention of obesity and inflammatory qualities. This study assessed the protective potential of SSPL on high-fat diet-induced obese C57BL/6J mice.

## Materials and methods

### Preparation of SSPL

SSPL is obtained from Jilin Agricultural Science and Technology College Edible Fungus Practice Center (Jilin, China). We prepared 100 mg/mL stocking suspended solution by diluting SSPL powder in distilled water for extraction storage. Meanwhile, due to the difference in body weight accompanied feeding weeks, the 650 mg/kg concentration of oral gavage volume was created on a weekly basis.

### Animal experimental protocol

Forty male C57BL/6J mice (age, 5 weeks old; starting body weight, 17–20 g) were obtained from the Jilin University (Changchun, China), and then mice were fed at a temperature condition of 23–25°C and a moisture content of 50–55% under circadian rhythm (12 h with light condition). Animals were routinely given special feed and drinking water.

Following a 1-week adaptation period, mice were used in a 12-week experiment. All animals were randomly split into four experimental groups (*n* = 10): control diet (CD); high-fat diet (HD); high-fat diet plus l-carnitine (HDL); high-fat diet plus sporoderm-broken spore powder of *Ganoderma lucidum* (HDG). Animals in the CD group were fed with a diet comprising 10% kcal with fat (NC, Normal-chow diet), whereas those in the HD group were fed with a meal containing 60% kcal with fat (HF, High-fat diet). The further treatment process initiated at the same time as the experimental diet feeding.

The HDL group was given oral administration of l-carnitine dosage at 250 mg/kg, and the HDG group was supplied with SSPL (650 mg/kg). Our ingredients were diluted in distilled water and orally offered a fixed volume of 100 μL once per day. The CD group was given an oral administration of the same volume of distilled water. Food intake was calculated once every 2 days. Body weight was weighed once a week. At the end of the 12th week, the animals were sacrificed after 12 h of overnight fasting. The blood, liver, and epididymal fat tissue were rapidly harvested and washed with cold sterile normal saline of tissue and were weighed and stored a –80°C until further assay.

### Serum biochemical parameters examination

Total cholesterol (TC), triglyceride (TG), high-density lipoprotein cholesterol (HDL-C), low-density lipoprotein cholesterol (LDL-C), aspartate aminotransferase (AST), and alanine aminotransferase (ALT) levels were automatically detected by assay kit. TC and TG assays were supplied from Jiancheng, Nan Jing, China. HDL-C and LDL-C assays were supplied from BioVision, Milpitas, CA, USA. Meanwhile, AST and ALT assays were supplied from Asan Pharmaceutical Co., Seoul, Korea. The serum concentrations of adiponectin and leptin as well as inflammatory-associated cytokines such as tumor necrosis factor-alpha (TNF-α), interleukin-6 (IL-6), and interleukin-1 (IL-1) were quantified using commercial ELISA kits (R&D Systems, Minneapolis, MN, USA) according to the manufacturer’s instructions.

### Hepatic lipids extraction and analysis

Hepatic lipids were extracted (20) and determined, and TC and TG were analyzed using the kits (Jiancheng, Nan Jing, China).

### Histopathological analysis

Hepatic tissue and epididymal fat were preserved in a 10% formalin (Abcam, USA) solution overnight before being embedded in paraffin (Thermo Fisher Scientific, USA). 4 µm slices were prepared and subsequently deparaffinized with the xylene (Sigma, St Louis, USA) and concentration gradient of ethanol and rehydrated using deionized H_2_O for two washes of 5 min. Furthermore, we stained with hematoxylin and eosin (H&E, Harris Hematoxylin and Eosin Y; Sigma, St Louis, USA) (21), the H&E-stained slides were inspected using a Leica Microsystems CMS GmbH (Wetzlar, Germany), and morphological pictures were analyzed using the SIS 3.2 software (Soft-Imaging System).

### Real-time polymerase chain reaction analysis

The expression of the roles of lipid metabolism and inflammation-related genes in the epididymal fat and liver tissue was determined by a real-time polymerase chain reaction (RT-PCR) assay. Total RNA was extracted from tissues utilizing TRIzol (Invitrogen, Carlsbad, CA). The isolated total RNA was counted, and cDNA was generated using the PrimeScript RT Master Mix (Takara, Kyoto, Japan). RT-PCR was conducted using an SYBR green qPCR mix (Toyobo, Osaka, Japan). The primer sequences used were obtained from PrimerBank and listed in Table 1.

**Table 1:** Primer sequence of the genes

Gene name	Primers	Sequence (5’-3’)
SREBP-1c	Forward	5’-AAGCAAATCACTGAAGGACCTGG-3’
	Reverse	5’-AAAGACAAGGGGCTACTCTGGGAG-3’
FAS	Forward	5’-AGGGGTCGACCTGGTCCTCA-3’
	Reverse	5’-GCCATGCCCAGAGGGTGGTT-3’
ACC	Forward	5’-CCAACATGAGGACTATAACTTCCT-3’
	Reverse	5’-TACATACGTGCCGTCAGGCTTCAC-3’
PPARα	Forward	5’-GGATGTCACACAATGCAATTCGCT-3’
	Reverse	5’-TCACAGAACGGCTTCCTCAGGTT-3’
CPT-1	Forward	5’-AAAGATCAATCGGACCCTAGACA-3’
	Reverse	5’-CAGCGAGTAGCGCATAGTCA-3’
PPARγ	Forward	5’-GATGACAGCGACTTGGCAAT-3’
	Reverse	5’-TGTAGCAGGTTGTCTTGAATGT-3’
Leptin	Forward	5’-TGACACCAAAACCCTCATCA-3’
	Reverse	5’-AGCCCAGGAATGAAGTCCA-3’
Adiponectin	Forward	5’-AAGGAGATGCAGGTCTTCTTGGT-3’
	Reverse	5’-CACTGAACGCTGAGCGATACAT-3’
ACOX1	Forward	5’-TATTCGGCTATGACTGGGCACA-3’
	Reverse	5’-GATGGATACTTTCTCGGCAGGA-3’
TNF-α	Forward	5’-ATGGCCCAGACCCTCACA-3’
	Reverse	5’-TTGCTACGACGTGGGCTACA-3’
IL-6	Forward	5’-GCTTAATTACACATGTTCTCTGGGAAA-3’
	Reverse	5’-CAAGTGCATCATCGTTGTTCATAC-3’
IL-1β	Forward	5’-GACCTTCCAGGATGAGGACA-3’
	Reverse	5’-AGCTCATATGGGTCCGACAG-3’
β-actin	Forward	5’-AGCCTTCCTTCTTGGGTATGG-3’
	Reverse	5’-CACTTGCGGTGCACGATGGAG-3’

### Statistical analysis

All data were expressed as mean ± SD (standard deviation, SD, was shown by error bars). To examine statistical differences, data were analyzed using one-way ANOVA in SPSS (version 23.0; SPSS, Inc., Chicago, IL, USA). The substantial variations among the groups were assessed by the Duncan’s multiple range test (*P* < 0.05), and values with various superscript letters (a, b, c, and d).

## Results

### Effect of SSPL on body weight, tissue weight, and food intake

**Fig. 1:**
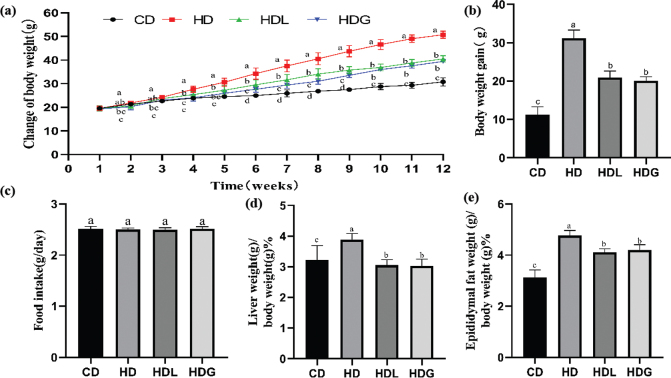
Effect of SSPL on body weight, tissue weight, and food intake in experimental mice. (a) Growth curve during the experimental period, (b) body weight gain, (c) food intake, (d) liver weight (g)/body weight (g) %, and (e) epididymal fat weight (g)/body weight (g) %. CD, control diet; HD, high-fat diet; HDL, high-fat diet plus l-carnitine; HDG, high-fat diet plus sporoderm-broken spore powder of *Ganoderma lucidum*. Data were assessed by ANOVA (Ducan’s test). Means with different superscript letters (a, b, c, and d) are significantly different from each other (*P* < 0.05). ‘a’ stands for the highest value, while ‘d’ stands for the lowest.

As seen in Fig. 1A, the basal body weight was unremarkably diverse among all the groups. The body mass of mice in the HD group was obviously increased than the mice in the CD group. However, treatment groups demonstrated a considerable decrease in body weight relative to the HD group. Substantial changes in body weight between the HD group and treatment groups (HDL and HDG) were found since the 4th week. After a 12-week experiment, the body weight growth of the HD group was evident, in contrast to the body weight gain of the HDL and HDG groups, which was greatly reduced (Fig. 1B). Noticeable changes were not shown in food consumption among all these groups (Fig. 1C). As demonstrated in Fig. 1D and 1E, the masses of the liver and epididymal fat versus body weight were significantly greater in the HD group than in the CD group. Moreover, HD-induced rise in liver weight was abolished in the HDL and HDG groups. The epididymal fat weight was dramatically lowered in the HDL and HDG groups in comparison to the HD group, and also even between HDL and HDG group that no significant differences were further observed.

### Effects of SSPL on lipid parameters of serum and liver

As indicated in Table 2, the HD group mice exhibited considerably greater levels of serum TC, TG, LDL-C, AST, and ALT than the CD group, whereas the HDL and HDG groups showed a huge reduction in levels as compared with the HD group. In contrast, HD group mice exhibited a considerably lower level of HDL-C than the CD group; likewise, the HDL and HDG groups demonstrated a notable recovery of HDL-C level relative to the HD group.

**Table 2:** Lipid parameters in serum and liver tissue

Groups	CD	HD	HDL	HDG
Serum (mg/dL)				
TC	93.60 ± 2.42^c^	194.24 ± 5.46^a^	127.13 ± 3.36^b^	129.35 ± 1.93^b^
TG	40.50 ± 0.80^c^	92.97 ± 3.24^a^	58.03 ± 1.05^b^	59.82 ± 1.64^b^
HDL-C	102.34 ± 7.86^a^	49.54 ± 1.48^d^	95.06 ± 3.32^b^	89.39 ± 6.59^c^
LDL-C	48.52 ± 0.98^c^	109.69 ± 1.92^a^	62.56 ± 1.5^b^	63.46 ± 1.25^b^
AST (IU/L)	49.28 ± 1.62^c^	101.35 ± 1.86^a^	66.35 ± 1.79^b^	63.60 ± 2.09^b^
ALT (IU/L)	42.16 ± 1.99^c^	89.22 ± 2.52^a^	72.29 ± 2.18^b^	69.32 ± 1.57^b^
Liver (mg/g)				
TC	1.44 ± 0.32^b^	3.52 ± 0.34^a^	1.56 ± 0.15^b^	1.59 ± 0.14^b^
TG	11.86 ± 0.94^c^	31.23 ± 1.24^a^	21.78 ± 1.71^b^	22.27 ± 0.72^b^

TC, total cholesterol; TG, triglycerides; HDL-C, high-density lipoprotein cholesterol; LDL-C, low-density lipoprotein cholesterol; AST, aspartate aminotransferase; ALT, alanine aminotransferase; CD, control diet; HD, high-fat diet; HDL, high-fat diet plus l-carnitine; HDG, high-fat diet plus sporoderm-broken spore powder of *Ganoderma lucidum*. Data were assessed by ANOVA (Ducan’s test). Means with different superscript letters (a, b, and c) are significantly different from each other (*P* < 0.05). ‘a’ stands for the highest value, while ‘c’ stands for the lowest.

Additionally, the HD group mice exhibited considerably greater levels of hepatic TC and TG than the CD group, whereas HDL and HDG groups showed a huge reduction in levels as compared with the HD group.

### Effect of SSPL on serum adipokines

**Fig. 2:**
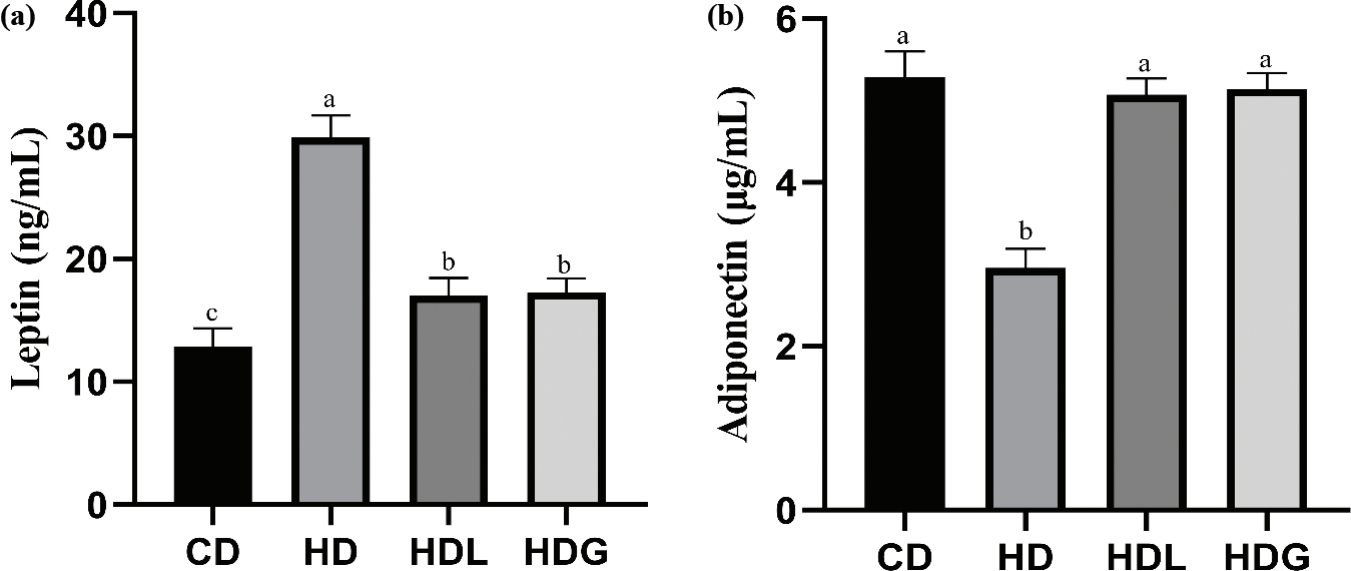
Effect of SSPL on serum adipokines. (a) Leptin level of serum and (b) adiponectin level of serum. CD, control diet; HD, high-fat diet; HDL, high-fat diet plus l-carnitine; HDG, high-fat diet plus sporoderm-broken spore powder of *Ganoderma lucidum*. Data were assessed by ANOVA (Ducan’s test). Means with different superscript letters (a, b, and c) are significantly different from each other (*P* < 0.05). ‘a’ stands for the highest value, while ‘c’ stands for the lowest.

As seen in Fig. 2, the HD group mice had a significantly higher level of leptin and a significantly lower level of adiponectin as well, and the HDL and HDG groups showed a significant reversal in levels of leptin and adiponectin compared with the HD group.

### Effect of SSPL on inflammatory cytokine of serum

**Fig. 3:**
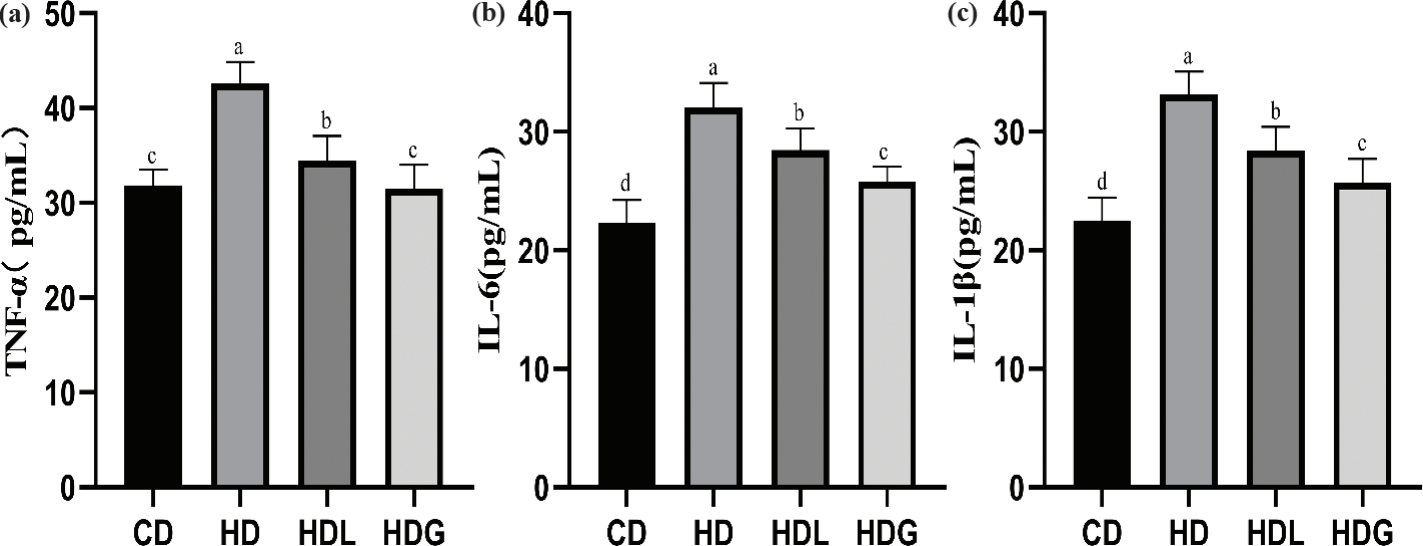
Effect of SSPL on serum inflammatory cytokine. (a) TNF-α level of serum, (b) IL-6 level of serum, and (c) IL-1β level of serum. CD, control diet; HD, high-fat diet; HDL, high-fat diet plus l-carnitine; HDG, high-fat diet plus sporoderm-broken spore powder of *Ganoderma lucidum*. Data were assessed by ANOVA (Ducan’s test). Means with different superscript letters (a, b, c, and d) are significantly different from each other (*P* < 0.05). ‘a’ stands for the highest value, while ‘d’ stands for the lowest.

Assessment of serum biological indicators (Fig. 3) revealed that the levels of TNF-α, IL-6, and IL-1β in serum were considerably elevated in the HD group in comparison with the CD group. Furthermore, the HDL and HDG groups indicated markedly lower TNF-α, IL-6, and IL-1β levels relative to the HD group.

### Effect of SSPL on liver and epididymal fat tissue

**Fig. 4:**
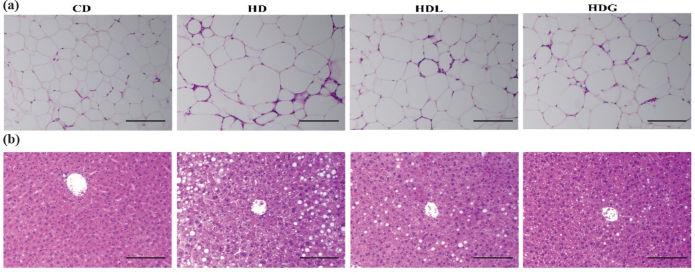
Histopathological analysis of epididymal fat tissue and liver in mice. (a) Epididymal fat tissue section (20×) by H&E staining and (b) liver section (20×) by H&E staining. CD, control diet; HD, high-fat diet; HDL, high-fat diet plus l-carnitine; HDG, high-fat diet plus sporoderm-broken spore powder of *Ganoderma lucidum*.

H&E staining was used to confirm the pathological alterations in epididymal fat and liver tissue (Fig. 4). The HD group adipose tissue showed significant hypertrophic manifestation compared with the CD group. In the HDL and HDG groups, the size of adipose tissue was dramatically smaller than the HD group.

The histopathologic assessment demonstrated a considerable increase in characteristics of lipid vesicles formation in the liver of the HD group compared with the CD group. HD-induced lipid droplets accumulation was observed to reverse in the HDL and HDG groups.

### Effects of SSPL on epididymal fat and liver lipid metabolism-related genes

**Fig. 5:**
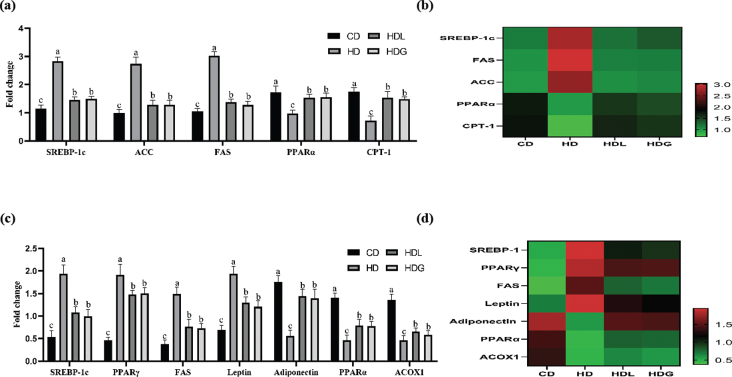
Effects of SSPL on adipose and liver tissue lipid metabolism-related genes of mice. (a) Relative mRNA levels of genes related to adipogenesis and lipid oxidation in epididymal fat tissue, (b) heat-maps for involved genes of epididymal fat tissue, (c) relative mRNA levels of genes related to adipogenesis and lipid oxidation in liver tissue, and (d) heat-maps for involved genes of liver fat tissue. CD, control diet; HD, high-fat diet; HDL, high-fat diet plus l-carnitine; HDG, high-fat diet plus sporoderm-broken spore powder of *Ganoderma lucidum*. Data were assessed by ANOVA (Ducan’s test). Means with different superscript letters (a, b, and c) are significantly different from each other (*P* < 0.05). ‘a’ stands for the highest value, while ‘c’ stands for the lowest.

In the epididymal fat tissue (Fig. 5A), the levels of SREBP-1c, FAS, and ACC, these adipogenesis-associated genes, were considerably increased in the HD group than the CD group. In the HDL and HDG groups, the expression of these genes was significantly attenuated relative to the HD group. The HD group exhibited the downregulated lipid oxidation-related mRNA expression of PPARα and CPT-1, whereas the HDL and HDG groups showed higher PPARα and CPT-1 levels more significantly than the HD group. In the liver tissue (Fig. 5C), the levels of adipogenesis-associated genes such as SREBP-1c, PPARγ, FAS, as well as leptin expression were higher in the HD group compared with the CD group. In contrast, these genes were downregulated in the HDL and HDG groups relative to the HD group. Moreover, in the HDL and HDG groups, adiponectin level was significantly increased compared with the HD group. The HD group exhibited the downregulated lipid oxidation mRNA expression of PPARα and ACOX1, whereas the HDL and HDG groups showed highest PPARα and ACOX1 levels more significantly than the HD group. Heat-map analysis also proved the corresponding results (Fig. 5B, 5D).

### Effects of SSPL on epididymal fat and liver tissue inflammation-related genes

**Fig. 6:**
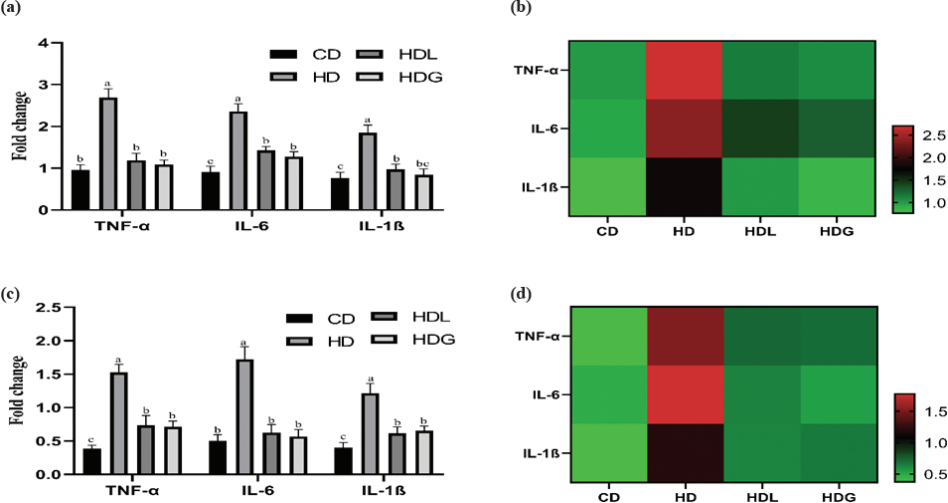
Effects of SSPL on adipose tissue pro-inflammatory-related genes of mice. (a) Relative mRNA levels of genes-related pro-inflammatory related in adipose tissue, (b) heat-maps for involved genes in adipose tissue, (c) relative mRNA levels of genes-related pro-inflammatory related in liver tissue, and (d) heat-maps for involved genes in liver tissue. CD, control diet; HD, high-fat diet; HDL, high-fat diet plus l-carnitine; HDG, high-fat diet plus sporoderm-broken spore powder of *Ganoderma lucidum*. Data were assessed by ANOVA (Ducan’s test). Means with different superscript letters (a, b, and c) are significantly different from each other (*P* < 0.05). ‘a’ stands for the highest value, while ‘c’ stands for the lowest.

The mRNA levels of TNF-α, IL-6, and IL-1β (pro-inflammatory genes) in adipose and liver tissue were inspected (Fig. 6). The HD group significantly upregulated mRNA expression of these inflammation-related genes (TNF-α, IL-6, and IL-1β) over that of the CD group. Compared with the HD group, the HDL and HDG groups had significantly downregulated pro-inflammatory mRNA expression of TNF-α, IL-6, and IL-1β. Heat-map analysis also proved the corresponding results.

## Discussion

Obesity is worldwide among the major health issues, and its complications disturb a massive of the population globally. A high BMI is associated with an increased risk of developing a variety of chronic conditions (22). Therefore, dietary alterations, increased physical activity, and lifestyle changes have become critical strategies in preventing and treating obesity. *Ganoderma lucidum* has a high concentration of active ingredients, including polysaccharides and triterpenoids (23, 24). Previous study has shown that *Ganoderma lucidum* extracts are widely used in the prevention of obesity due to the presence of numerous functional bioactive constituents that may interact with signaling pathways implicated in obesity and insulin resistance (25). Previous research has demonstrated that the preventive effect can significantly lower the body weight, liver, and fat index of mice, as well as significantly ameliorate hyperlipidemia (26). This study demonstrates unremarkable differences in dietary intake in all groups. While, after 12 weeks of oral gavage administration with SSPL, the weight gain of C57BL/6J mice was significantly lower than that of the HD group, likewise in the HDG group, fat index of liver and epididymis was decreased significantly.

A significant increase in serum lipid levels, decreased adiponectin levels, and elevated leptin levels are essential characteristics of obese patients (27, 28). PGL can improve lipid metabolism of Japanese big-ear white rabbits such as downregulated to TC and TG (29). In this study, compared with the HD group, supplementation with SSPL improved serum lipid levels such as TC, TG, HDL-C, LDL-C, AST, and ALT. The HDG group showed a suppressed leptin concentration and increased adiponectin concentration compared with the HD group. The liver is the main organ for the formation of new lipids that produce triglycerides through an enzymatic reaction (30). HFD consumption induced hepatic lipid metabolism dysfunction, and increased TC and TG synthesis (31). PGL has the effect of improving liver steatosis (14). Our present study notably exhibits hepatic TC, and TG levels were significantly decreased in the HDG group compared with the HD group.

Over time, a high-fat diet is connected with a rise in body weight and fat mass, as well as fat accumulation in organs such as the heart, liver, and blood vessels, all of which contribute to weight gain (32). The HDL and HDG groups exhibited less lipid accumulation relative to the HD group in the liver. Also, we evidenced a reduction in the number of lipid droplets in H&E-stained liver tissue sections. Furthermore, the size of adipose tissue was improved in the HDG group. A previous study has literarily announced that *Ganoderma lucidum* reduced size of adipose tissue and lipid accumulation, consistent with our findings (26). The consumption of a high-fat diet may result in low-grade inflammation and liver damage, as well as an increase in pro-inflammatory cytokines such as TNF-α, IL-6, and IL-1β (33). *Ganoderma lucidum* showed significant anti-inflammatory activity in the prevention and treatment of a number of inflammatory disorders (34). Ganoderma triterpenes have been proven to have anti-inflammatory properties in previous research (35). PGL could alleviate D-gal-induced cognitive impairment through the mechanism of suppressing inflammation and metabolic disturbances (36). In our study, the concentration of TNF-α, IL-6, and IL-1β in serum decreased significantly in the HDL and HDG groups and obviously increased in the HD group. The same result has also been shown in genes expression. Our study showed that the HDG group improved the inflammatory conditions of liver and epididymal fat by lowering pro-inflammatory cytokine levels, and therefore, advance prevented obesity.

Higher levels of SREBP-1c, PPARγ, FAS, and ACC are characteristics of obesity. SREBP-1c possesses a critical role in the regulation of FAS synthesis. The SREBP-1c/FAS pathway was greatly enhanced in obese mice, promoting hepatic steatosis (12, 37). ACC catalyzes the production of malonyl-CoA, which is the main component of lipogenesis formation (38). Phosphorylated ACC activates the enzyme and facilitates the oxidation of free fatty acids (39). ACC is a lipase regulated by SREBP-1c (40). Recent study has demonstrated that the polysaccharide peptide from *Ganoderma lucidum* suppressed fatty acid production by suppressing the expression of SREBP1c, FAS, and ACC and decreasing the accumulation of lipid droplets (41). PPARγ is a ligand-activated nuclear receptor constitutively expressing and playing an important role in serum lipids regulation (42). A previous study reported that supplement extracts from medicinal mushroom *Ganoderma lucidum*, such as triterpenes and polysaccharides, inhibited adipocyte differentiation through the suppressed expression of adipogenic transcription factors such as PPARγ, SREBP-1c, and FAS (43). PPARα activation leads to the transcription of CPT-1, a target gene that is crucial to β-oxidation as it allows the fatty acid to reach the mitochondrial matrix (44). Adiponectin contributes to β-oxidation by mediating the phosphorylation of AMP-activated protein kinase (AMPK) and activating PPARα (45). As known, PPARα might improve fatty acid oxidation through the regulation of key enzymes such as CPT-1 and ACOX1 (46). This study shows that the HDG group downregulated the expression of lipogenesis genes in epididymal fat, such as SREBP-1c, FAS, and ACC. However, the HDG group upregulated the expression of lipid oxidation genes in epididymal fat, such as PPARα and CPT-1. In our study, compared with the HD group, the SREBP-1c, PPARγ, FAS, and leptin in the liver were significantly reduced in the HDG group. Compared with the HD group, the adiponectin, PPARα, and ACOX1 in the liver were significantly increased in the HDG group. Therefore, the triterpenes and polysaccharides-rich SSPL appear to be responsible for the alteration in lipid accumulation.

## Conclusion

In this study, SSPL administration to obese mice fed with a high-fat diet demonstrated anti-obesity and anti-inflammatory benefits via modulation of inflammatory cytokines, lipogenesis, and lipid oxidation genes. Additionally, biochemical indicators of the serum and liver, such as TC, TG, HDL-C, LDL-C, AST, ALT, leptin, adiponectin, TNF-α, IL-6, and IL-1β, improved in the HDG group.

## Conflicts of interest

The authors declare no conflict of interest.

## Funding

This research was funded by ‘Jilin Agriculture Science and Technology University’ Ph.D. fund (20217003). This work was supported by Jilin Agriculture Science and Technology University and Food Science and Engineering Key Discipline Cultivation Project (2019X2001). This work was also supported by the Nutrition and Healthy Food Research Team of Jilin Agriculture Science and Technology University.

## Author contributions

Conceptualization, Methodology, Validation, Writing – original draft, Writing – review & editing, and Project administration: B.Z.; Software, Validation, and Writing – review & editing: C.P.; Formal analysis: J.-Y.Z.; Validation and Resources: Y.F.; Supervision and Project administration: F.-L.L. All authors have read and agreed to the published version of the manuscript.

## Institutional review board statement

This study was conducted with the approval of the Animal Ethics Committee of Jilin Agriculture Science and Technology University (approval number: 2021012), and the mice were accepted as animals in compliance with the national standard before the introduction.

## Informed consent statement

Not applicable.
